# Supplementary material for: Selective auditory attention modulates cortical responses to sound location change for speech in quiet and in babble
Source: PLoS One. 2023 Jan 13;18(1):e0268932. doi: 10.1371/journal.pone.0268932 (PMC9838839; doi:10.1371/journal.pone.0268932)
Supplement: Attachment — Submitted filename: PLOS Review.docx [file pone.0268932.s002.docx]

Review of “*Selective auditory attention modulates cortical responses to sound location change for speech in quiet and in babble*”

Abstract:

The abstract was concise but more simplified results are needed. For example, I did not understand the sentence, “*However, the effect of the two background environments diverges when considering the magnitude and direction of the location change, in which there was a clear influence of change vector in quiet but not in babble*.” What was the direction in which subject performed better? Or was the subject able to change location more easily in quiet? The authors may also want to be more descriptive of cortical object formation (line 29) and what it means in respect to the cocktail party effect.

Introduction:

The premise of manuscript is setup nicely in the first three paragraphs. Previous literature supports this current work and show a need for better understanding how auditory object formation is affected by the interaction of spatial location and attention. In addition, the authors highlight the use of speech as evoking stimuli and background noise to make this experiment even more ecologically valid. My only critique of the introduction is the wording of the last paragraph. The goals and hypotheses need to be clearly outlined. Keep it simple. Also, what were the main independent variables? From my review it seems like there are three variables of interest: location, attention, and background noise.

Methods:

The procedural and electrophysiologic methodology is well defined and clear. It is obvious that an electrophysiology researcher is the primary author as someone could easily read this and replicate the study. I would have liked to see more details regarding the specific measures of the components (N1-P3). For example, were amplitude, latency, or even area measures considered? However, there is one large problem with the babble masker, they in eight foreign languages? This needs to be discussed as foreign language maskers have been shown to be basically energetic maskers especially if the talkers exceed 6. More discussion of why the authors chose foreign language maskers is warranted. The final and major critique of the methods section is the lack of statistical and power analysis section. What were the planned comparisons? This goes back to the introduction as there does not seem to be a clear analysis strategy. How were the conditions compared and what statistics were conducted? Was a linear-mixed effect model used to address the repeated measures? How many conditions and was there a correction factor for number of analyses? Also, is the study adequately powered for only 18 subjects? A brief power analysis needs to be documented to show that results are interpretable given the low n.

Results:

The results show some interesting findings, but again it seems like these analyses were exploratory in nature. For example, why was the epoch window set to 2.0s? If the N1 and P3 were components of interest why show the waveform to 2.0s? It seems like the major findings are the difference between active and passive (Active = larger amplitude), quiet and babble (quiet = larger amplitude), and the larger magnitude for the rightward direction of tart identification. Considering the first two results should have been hypothesized, the rightward direction is fascinating. A few more follow-up questions:

1. Why were only correct trials counted? What if the object was not formed? This is valuable information that could provide more insight in the babble condition especially.
2. The authors mention the P3 component in the passive condition? This is a fundamental flaw and needs to be reworded as the P3 is only elicited in an active condition. A more detailed description of the primary components of interest is warranted in the methods.
3. Minor - Paired sample t-test alpha 0.5 (line 195)? I believe this should be 0.05.

Discussion:

The discussion identified the major findings and provided a rationale for results. The N1 was modulated by attention?

What latency analysis did you find (Line 259)?

P3 is an automatic process (Line 280)? I completely disagree with this statement.
