# Supplementary material for: Selective auditory attention modulates cortical responses to sound location change for speech in quiet and in babble
Source: PLoS One. 2023 Jan 13;18(1):e0268932. doi: 10.1371/journal.pone.0268932 (PMC9838839; doi:10.1371/journal.pone.0268932)
Supplement: Attachment — Submitted filename: Ozmeral&Menon_PLOS Review Response.docx [file pone.0268932.s003.docx]

Reviewer #1: Review of “Selective auditory attention modulates cortical responses to sound location change for speech in quiet and in babble”

Overall, I found the manuscript to be intriguing and detailed. However, the “exploratory” nature of the study needs to either be explicitly identified or the following changes made:
1. Description of the ERPs of interest and why/how they were elicited? In addition, what do they mean in relation to auditory attention and object formation?
2. Primary hypotheses and statistical analyses stated. The planned analyses are not stated in any section leading the manuscript to have too much of an “exploratory” feel for this journal.

We thank the reviewer for your important comments. The manuscript has undergone targeted revisions to directly address each of your concerns. Importantly, we have stated the hypotheses more clearly, and the statistical analyses that followed are more appropriately motivated and thoroughly described. Each of the additional comments below are also addressed. Thank you again.

Abstract:

The abstract was concise but more simplified results are needed. For example, I did not understand the sentence, “*However, the effect of the two background environments diverges when considering the magnitude and direction of the location change, in which there was a clear influence of change vector in quiet but not in babble*.” What was the direction in which subject performed better? Or was the subject able to change location more easily in quiet? The authors may also want to be more descriptive of cortical object formation (line 29) and what it means in respect to the cocktail party effect.

We made some changes to the abstract. We changed line 29 to better tie it to cocktail party listening: “… into the cortical representations of auditory scene analysis.” We also revised the section regarding the different effects seen with and without babble present to be more specific: starting at “However, …” (line 38)

Introduction:

The premise of manuscript is setup nicely in the first three paragraphs. Previous literature supports this current work and show a need for better understanding how auditory object formation is affected by the interaction of spatial location and attention. In addition, the authors highlight the use of speech as evoking stimuli and background noise to make this experiment even more ecologically valid. My only critique of the introduction is the wording of the last paragraph. The goals and hypotheses need to be clearly outlined. Keep it simple. Also, what were the main independent variables? From my review it seems like there are three variables of interest: location, attention, and background noise.

The final paragraph in the intro has been revised to more clearly state hypothesis. Explicit reference to IVs is now stated in a statistical approach section of the methods.

Methods:

The procedural and electrophysiologic methodology is well defined and clear. It is obvious that an electrophysiology researcher is the primary author as someone could easily read this and replicate the study. I would have liked to see more details regarding the specific measures of the components (N1-P3). For example, were amplitude, latency, or even area measures considered? However, there is one large problem with the babble masker, they in eight foreign languages? This needs to be discussed as foreign language maskers have been shown to be basically energetic maskers especially if the talkers exceed 6. More discussion of why the authors chose foreign language maskers is warranted. The final and major critique of the methods section is the lack of statistical and power analysis section. What were the planned comparisons? This goes back to the introduction as there does not seem to be a clear analysis strategy. How were the conditions compared and what statistics were conducted? Was a linear-mixed effect model used to address the repeated measures? How many conditions and was there a correction factor for number of analyses? Also, is the study adequately powered for only 18 subjects? A brief power analysis needs to be documented to show that results are interpretable given the low n.

Thank you for the critique of the methods. With respect to the babble, we agree that it would be primarily an energetic masker, and we now point that out in the section on materials with an appropriate reference. We also have added a new section “Analyses and Statistical Approach” where we address the reviewer’s other feedback. Specifically, we describe the extraction method of the AERP peaks, the relevant condition comparisons, and the statistical approach as they pertain to the hypotheses. Some of this information was integrated in the Results section previously but is now consolidated here for clarity.

Results:

The results show some interesting findings, but again it seems like these analyses were exploratory in nature. For example, why was the epoch window set to 2.0s? If the N1 and P3 were components of interest why show the waveform to 2.0s? It seems like the major findings are the difference between active and passive (Active = larger amplitude), quiet and babble (quiet = larger amplitude), and the larger magnitude for the rightward direction of tart identification. Considering the first two results should have been hypothesized, the rightward direction is fascinating.

Thank you for the questions. The epoch window was set to 2 seconds because this was roughly the amount of time that the stimuli were at each location before a change (not including catch trials). As for the rightward direction, we now include a paragraph discussing this at the end, though we admit that because we’re not using source analyses, and we did not intentionally investigate this phenomenon, our explanation is purely speculative.

A few more follow-up questions:

1. Why were only correct trials counted? What if the object was not formed? This is valuable information that could provide more insight in the babble condition especially. The reviewer brings up an interesting point, but because accuracy was above 95% for all subjects, the lack of statistical power for the incorrect trials would not be there, so these were excluded.
2. The authors mention the P3 component in the passive condition? This is a fundamental flaw and needs to be reworded as the P3 is only elicited in an active condition. A more detailed description of the primary components of interest is warranted in the methods. Thank you, this has been fixed, including the section added (mentioned above)
3. Minor - Paired sample t-test alpha 0.5 (line 195)? I believe this should be 0.05. Fixed

Discussion:

The discussion identified the major findings and provided a rationale for results. The N1 was modulated by attention?

What latency analysis did you find (Line 259)?

This is referring to the average latency relative to what’s found in the literature typically for onsets or acoustic change components that might occur at 100 ms and 200 ms after onset for N1 and P2. Our latencies were closer to 150 and 250 ms.

P3 is an automatic process (Line 280)? I completely disagree with this statement.

This was poorly worded before – the point is that a top-down process may have been unavoidable despite instruction to ignore the stimuli. This line now says, “this response may reflect an exogenous capture of attention”.

Reviewer #2: Review of Ozmeral and Palandrani, “Selective auditory attention…”

The manuscript describes a study of cortical EEG potentials evoked by changes in the location of a speech stream. The factors under study include the influence of background sound (quiet vs speech babble), the magnitude of spatial change, the attentional state of the participant (attend left, right, or to irrelevant visual), and the alignment of spatial change with attended (target) location, when applicable. The results demonstrate strong modulation of potentials by attention and by magnitude of spatial change. Effects of background sound were muted but could be extracted in a detailed analysis of amplitude for particular pairs of pre/post switch locations.

In general the manuscript is well written. The study appears clearly organized and correctly executed. Results are analyzed in a (mostly) straightforward manner that is justified, and the conclusions are reasonable.

I have no significant concerns about publication of this manuscript, although there are elements which are not very clear in the current form. These should be addressed or omitted.

General comments

My main concerns are that the analyses depicted in Fig 6 and Fig 7 are not as clear as they could be. Why focus on the time window around 550 ms?

Thank you for the question. As we now state in more detail in the intro, we had particular hypotheses regarding the effects of attention, and previous work indicated that this would be particularly noticeable at late-latency potentials. This is the reason for that time window.

For Fig 6: Why was it necessary to use a heat map for the pre/post location data in Fig 6? Does Fig 6 demonstrate anything interesting other than greater response for targets (i.e. post-switch location matches attended location)? The color scale of Fig 6 makes the babble results appear qualitatively different from the quiet, but comparing Figs 3 and 5 it looks like the results are similar, just modestly reduced amplitude in babble.

The point of Fig 6 was to demonstrate the change in responses as a function of the pre-switch location. These data are collapsed in Fig 7, but we thought this would be a valuable intermediate step when describing where Fig 7 came from. This is now stated explicitly in the text.

For Fig 7: Would like to see an independent assessment of “target” vs non-target spatial change in this analysis.

It’s not clear what the reviewer means by this comment. The data in Figure 7 derive from all spatial changes of the speech (target) and are not dependent on whether the speech was at a target location or non-target location.

Also the text’s description of the x-axis value is not clear. (I think it’s a simple enough idea, just not explained clearly; see specific comments below) Perhaps it would be useful to define a new term like “spatial change” or “signed shift magnitude” and define it quantitatively in the text?

Thank you for this suggestion. We have changed the x-axis label to be Spatial Change (°) and we have indicated in the text how this value was derived.

Finally, where is the discussion of the apparent asymmetry in the data (larger responses for rightward shifts in attend right but not leftward shifts during attend left? )

Thank you. We now include a new paragraph discussing this effect at the end of the discussion.

Specific:

Line 43: what is meant by “shunted”? This word is not longer used in the revised abstact

Line 183: it would be useful to know the effect of the button press itself, or of the “respond to a target” event. For example, a difference between passive and “attend” conditions is that only the latter have targets, so successful detection can only occur in those conditions. Thus, the evoked potential differences may not have anything to do with attention, per se, (or spatial attention in particular) but could mainly reflect the occurrence of “target detection” / “yes button” events.

The reviewer brings up an interesting point, which is why the analyses focus so heavily on the difference between the Attend Left versus the Attend Right conditions that have a button press for all trials. One way to get at the effect of the button press	would be to measure differences for the catch trials between Attend conditions and Passive conditions. These are the trials along the diagonal in Fig 6. At least from glancing at that, it appears there are elevated responses in the attention conditions relative to the passive, indicated a heightened level of activity without the button press. Also, based on the time period that listeners respond following the change in location, it’s likely that the peaks seen at around 1.5 s are indeed related to the button response which is why we chose to spend less time analyzing that peak.

Line 185: explain how the presence of P3 indicates stimulus factors rather than response. I suspect there is an model-based assumption hiding in this reasoning.

Per reviewer 1, this line was removed. Our previous reasoning was that for the Passive condition, there were no overt responses, so the presence of a late-latency deflection could represent a stimulus-driven response; however, this is too speculative for the results. We do mention in the discussion now that it’s possible that this was due to some exogenous capture of attention.

Line 228: may be clearer to say larger responses for post-switch locations matching target (describing the experiment) rather than “column” (describing the figure).

Thanks, good point. This has been revised.

Line 232: This analysis is not very clearly described. I think it is as follows: all location-change events were collected and binned according to the absolute displacement (size and direction) of the change, without regard to the pre or post switch position. The average GFP amplitude is plotted against displacement in Fig 7. However, it should be noted that the analysis includes both target and non-target trials so it is not an independent assessment of the effects of stimulus location change.

Thank you for the suggested wording. We adopted most of it and it now reads as, “To measure the effect of magnitude and direction of the location changes shown in Figure 6, all location-change events measured around 550 ms were collected and binned according to the absolute displacement (size and direction) of the change, without regard to the pre or post switch position. The average GFP amplitude was plotted against this “spatial change” in Figure 7. It should be noted, however, that the analysis includes both target and non-target trials so it is not an independent assessment of the effects of stimulus location change.”

Line 482: I feel there is a lot missing from the caption for fig 7. What is a “spatial-change vector?” What do the different colored lines represent? Do the plots include all changes including both “yes” and “no” trials?

It was a little light on detail, wasn’t it? This has been remedied.

Fig 7: Some values of change are more likely to include target locations than others, although none are exclusively targets. How would this result change if only non-targets were plotted?

It’s an interesting question. We ran the reviewer’s suggested analysis by eliminating the trials that were “targets” for the Attend Left and Attend Passive conditions, and the result was the figure below. Clearly, the Passive had no change, but the rightward bias was reduced for the Attend Right in Quiet condition, especially at a spatial change of +60. Considering the paper revolves around the effect of attending to a spatial hemifield, however, we’re not sure how best to include this side analysis.
